# Fatal Outcome of Imatinib in a Patient with Idiopathic Hypereosinophilic Syndrome

**DOI:** 10.1155/2018/6291614

**Published:** 2018-03-26

**Authors:** Ashraf Abugroun, Ahmed Chaudhary, Gabriel Rodriguez

**Affiliations:** Advocate Illinois Masonic Medical Center, Chicago, IL, USA

## Abstract

Cytokine storm is a poorly explained clinical entity caused by an undesired and aggrandized immune system response leading to unregulated activation of the proinflammatory cascade, often contributing to multisystem organ failure and even death. Its potentially diverse etiologies and sepsis-like presentation have made it even more challenging to diagnose, and so far, no well-established treatment protocol has been proposed. Its association with certain medications, especially with monoclonal antibodies, has well been reported in literature. To the best of our knowledge, so far, no previous case of cytokine storm associated with imatinib has been reported. We herein present a case report of a 77-year-old male with a past medical history of hypereosinophilic syndrome who developed acute fatal cytokine storm following treatment with imatinib. This study highlights a life-threatening complication of the medication that may be underrecognized.

## 1. Introduction

Imatinib is a tyrosine kinase inhibitor that is used in various hematologic malignancies, including idiopathic hypereosinophilic syndrome (IHES) which is a myeloproliferative disorder characterized by sustained, nonreactive, unexplained persistent hypereosinophilia that commonly results in multiorgan dysfunction. This case highlights the development of a cytokine storm with severe uncontrolled systemic inflammatory response with a fatal outcome following the initiation of imatinib in a patient with IHES.

## 2. Case Presentation

A 77-year-old male with a history of IHES, COPD, CKD stage III, and active *Mycobacterium avium* complex (MAC) infection on treatment with rifampin, azithromycin, and levofloxacin was sent to the ER from oncology clinic for evaluation of progressive weakness, lethargy, and hyperkalemia.

**Figure 1:**
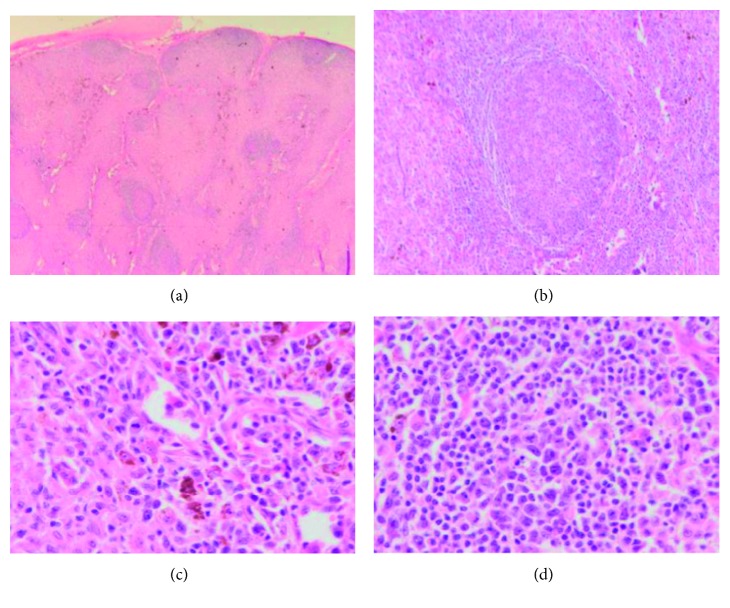
Lymph node biopsy. H&E stain. Low-power view (a). Paracortical (T-zone) hyperplasia (b). At higher magnification (c, d), this zone contains interdigitating dendritic cells, Langerhans cells, and melanin-containing histiocytes. There are also scattered immunoblasts in the paracortex of unknown significance.

**Figure 2:**
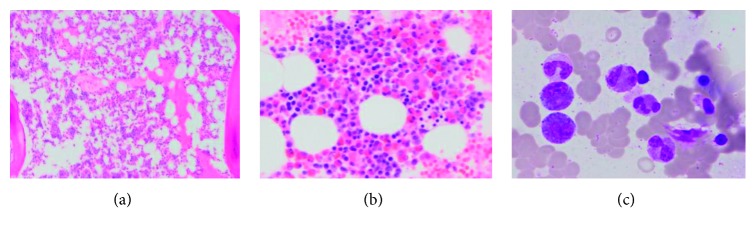
The bone marrow shows eosinophilia. Low-power H&E of the core (a), a high-power H&E from the clot section (b), and an oil immersion image from the aspirate (Giemsa stain) (c).

The patient had outpatient workup for unexplained hypereosinophilia. He underwent lymph node biopsy which showed no evidence of lymphoma (Figure 1). Peripheral blood flow cytometry showed myeloid and lymphoid cells with unremarkable immunophenotypic expression. Bone marrow biopsy showed eosinophilia that varied from approximately 25% in the aspirate smears to 60% in the core biopsy (Figure 2). The infiltrate of eosinophils consisted of eosinophilic myelocytes and mature eosinophils. There were no increase in blasts and no morphologic evidence of lymphoma. The patient had negative fluorescence in situ hybridization (FISH) results using a panel for hypereosinophilia containing probes for 4q12 (SCFD2, LNX, and PDGFRA) rearrangement, 5q32 (PDGFRB) rearrangement, 8p11.2 (FGFR1) rearrangement, and 9q34 and 22q11.2 (BCR/ABL1) rearrangement on a bone marrow specimen. He had normal cytogenetic studies and male-type karyotype. A final diagnosis of idiopathic hypereosinophilic syndrome was made. His disease was resistant to steroids and brief course of chemotherapy with methotrexate.

**Figure 3:**
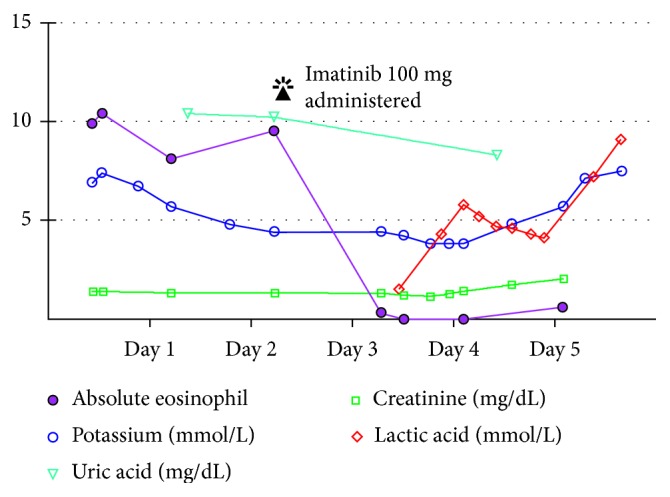
Timeline summarizes the laboratory changes on daily follow-up throughout admission. Rapid decline of eosinophil count from 9,500/mcl to zero levels within less than 24 hours from administration of imatinib is noted. Uric acid level slightly declined and potassium level remained stable except for the last day during which it raised progressively to the level of 7.5 mmol/L.

**Figure 4:**
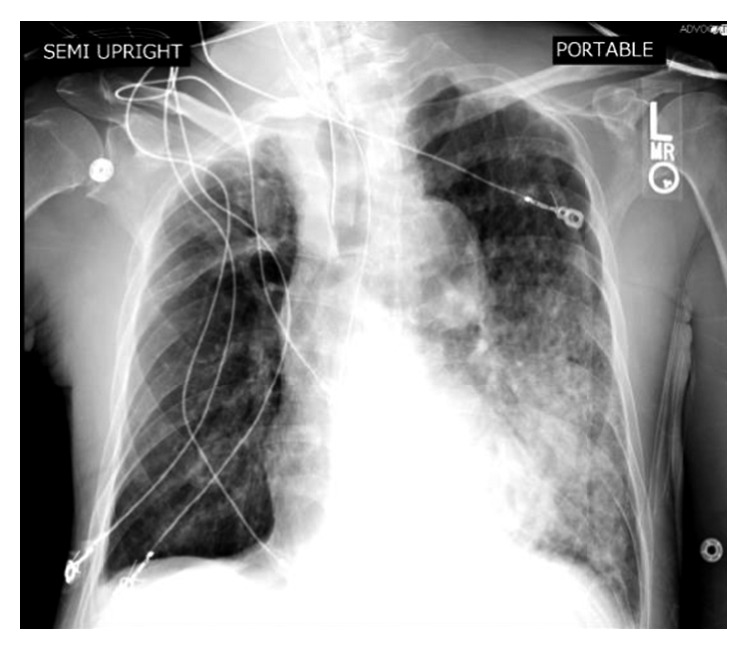
Chest X-ray showed patchy airspace infiltrates in the left lower lobe suggestive of pneumonia.

**Figure 5:**
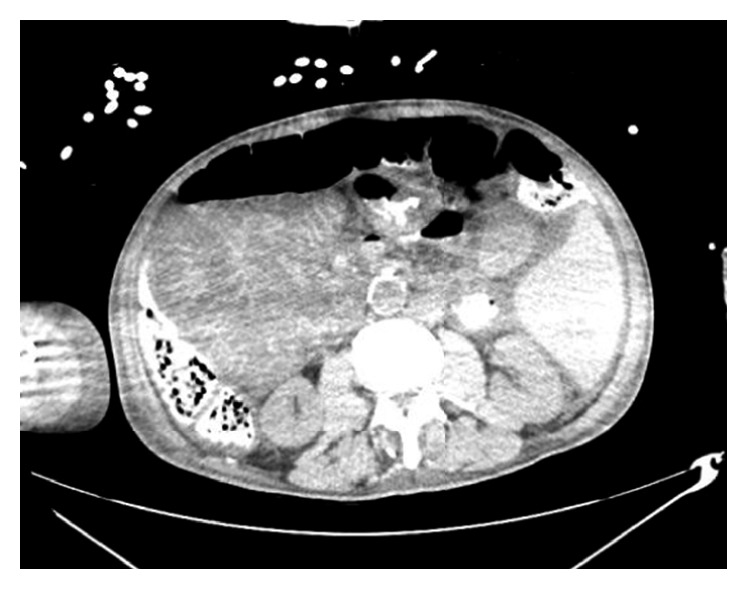
CT abdomen and pelvis showed prominent bowel loops with wall thickening, suggestive of bowel ischemia.

On his current admission, he was lethargic and cachectic. Vital signs were normal. Skin examination showed widespread erythroderma, scaling, and excoriations. Initial laboratory workup revealed potassium 6.9 mmol/L, creatinine 1.3 mg/dL, alkaline phosphatase 671 unit/L, and WBC 18,000 cells/mcL with 58% eosinophils. Hyperkalemia resolved on the third day of admission. Subsequently, a decision was made to start imatinib at a lower than recommended dose due to the high risk of developing tumor lysis syndrome. Shortly after initiation of imatinib, the patient developed acute restlessness, agitation, shortness of breath, and acute urinary retention. His condition rapidly deteriorated and progressed to acute respiratory failure requiring intubation and mechanical ventilation. He also developed distributive shock with hypothermia and nonanion gap metabolic acidosis. Absolute eosinophil count dropped dramatically from 9,500 cells/mcL to 0 cells/mcL within less than 24 hours following administration of imatinib (Figure 3). Given the dramatic decline in clinical condition, imatinib was immediately discontinued, and the patient was treated empirically with methylprednisolone and broad-spectrum antibiotics. The patient had normal echocardiogram and brain CT scan. On the next day, chest X-ray showed patchy airspace infiltrates in the left lower lobe suggestive of pneumonia (Figure 4). Blood cultures showed no microbial growth, and respiratory culture eventually grew multidrug-resistant organisms (MDROs) *Klebsiella pneumoniae* and moderate *Pseudomonas aeruginosa*. A CT abdomen and pelvis showed prominent bowel loops with wall thickening, suggestive of bowel ischemia (Figure 5). The patient condition dramatically declined and died following acute cardiac arrest on the sixth day of admission.

## 3. Discussion

According to the World Health Organization (WHO) classification, hypereosinophilic syndrome (HES) is classified into three distinct subsets caused by different pathogenic mechanisms including chronic eosinophilic leukemia caused by fusion protein (FIP1L1-PDGFRA) with tyrosine kinase activities, lymphoproliferative HES caused by clonal IL-5/Th2 lymphocyte-mediated hypereosinophilia, and idiopathic or myeloproliferative HES where no evidence of clonal hypereosinophilia is identified [1]. Patients with HES present with variable clinical manifestations related to the target organ involved by eosinophils. Although all body parts can be involved, the disease commonly involves the skin, heart, digestive tract, and nervous system [1]. The pathogenesis of HES is related to various molecular and cytogenetic abnormalities that ultimately result in overactivity of tyrosine kinase causing dysregulated clonal overproduction of eosinophils [2].

The goal of therapy in HES is to prevent eosinophil-induced progressive end-organ damage and irreversible complications of sustained hypereosinophilia. Available therapeutic options include corticosteroids, hydroxyurea, interferon-alpha, bone marrow transplant, and other cytotoxic and immunomodulatory agents [1, 3]. Due to low incidence of HES and increased heterogeneity of presenting symptoms, there are paucity of available data and limited numbers of studies on treatment options. Most studies on treatment are restricted to case reports and case series. The tyrosine kinase inhibitor imatinib was recently proposed as a rescue medication with high response rate among patients with HES refractory to medical therapy [4]. Imatinib was found to be particularly useful among patients with positive (FIP1L1-PDGFRA) phenotypes. In few cases, some reported therapeutic effect with the use of imatinib in idiopathic HES with negative (FIP1L1-PDGFRA) phenotypes [5–9]. The mechanism of action through which imatinib achieved success in these patients remains unknown [9].

Cytokine storm syndrome refers to massive release of cytokines from an undesired activation of the immune system by various factors that lead to uncontrolled systemic inflammatory response causing multiple organ dysfunction and often death. Cytokines are glycoproteins that are produced by leukocytes. They may have autocrine, paracrine, and endocrine actions, which explains both local and systemic response seen in cytokine storm. Under normal circumstances, cytokine causes monitored release of proinflammatory mediators, which not only helps the body mount response to any threat but also prevents any further injury to local tissue and helps in the process of healing. Contrarily, in cytokine storm, unregulated and robust activation of proinflammatory mediators aggrandized immune cellular lineages with enhanced activity, resulting in the unregulated cascade of acute inflammatory response. This all contributes to endothelial cell dysfunction that leads to increased capillary permeability/leak, which has been postulated as a mechanism for parenchymal injury and end-organ damage. As expected, organs susceptible to the worst injuries are the ones with stronger immune defense mechanisms, notably the lungs and gut [10].

Cytokine storm has been attributed to certain medications and treatments. Of note, monoclonal antibodies have long been associated with cytokine storm. In 2006, the first human clinical trial of TGN1412 monoclonal antibody to treat rheumatologic disease was conducted, and it was proposed that all 6 volunteers participated had fatal outcome from TGN1412-induced cytokine storm [11]. Alemtuzumab, another monoclonal antibody used in treatment of chronic lymphocytic leukemia, has been well known to be associated with cytokine release [12]. Unfortunately, no clear guidelines are available on how to approach cytokine storm. The primary goal of suggested treatments is to prevent end-organ damage and multiorgan failure through adequate resuscitative measure, use of broad-spectrum antibiotics, fluid resuscitation, and vasopressors if indicated. Anti-inflammatory medications including steroids and nonsteroidal medications have also been proposed but have been shown to have limited benefit/efficacy.

Imatinib, a monoclonal antibody, which acts as a tyrosine kinase inhibitor is FDA approved for treatment of hematological malignancies. It is considered to be a safe and effective drug and is on the WHO's list of essential medicines. Imatinib has well tolerability with a low incidence of toxicities [13]. Common side effects reported include nausea, diarrhea, visual disturbance, itchy rash, fluid retention, weight gain, and pancytopenia. On rare instances, some of the adverse effects reported with the use of imatinib include tumor lysis syndrome, hepatotoxicity, dermatological manifestations, left ventricular dysfunction, and cardiogenic shock [14–16]. Unexpectedly, our patient developed acute distributive shock and multiorgan failure immediately following the first dose of imatinib. The pathogenesis of such acute clinical decline is explained by the massive histamine release and cytokine storm, and this hypothesis is evident by the temporal relation between the initiation of imatinib therapy and the dramatic decline of eosinophilic count to zero within hours of therapy. The clinical presentation and laboratory data did not support a diagnosis of tumor lysis syndrome. His uric acid level dropped from the initial level of 10.4 gm/dl to 8.3 gm/dl following imatinib administration. Unfortunately, further laboratory tests including the level of plasma interleukins which could have further supported the diagnosis of cytokine storm were not performed.

Given the nonspecific presentation of cytokine storm, possibility of coexisting septicemia cannot be ruled; although the patient had left lower lobe infiltrates and positive respiratory cultures, it is worth mentioning that prior to imatinib administration, there was no clinical or radiological suspicion of the acute infectious process. Moreover, the patient had two blood cultures taken following the onset of distributive shock, and both did not show any bacterial growth. Though negative blood cultures cannot rule out septicemia as a cause of death, the timeline of events and the clinical, laboratory, and radiological findings that developed as a consequence of initiation of imatinib therapy pointed towards a massive cytokine storm (Figure 3).

## 4. Conclusion

In this case, imatinib caused severe acute distributive shock and multiorgan failure secondary to massive histamine release and cytokine storm evident by the temporal relation with initiation of therapy and dramatic decline of eosinophilic count to zero. Such an observation has not been documented in the past. This study highlights a life-threatening complication of the medication that may be underrecognized. HES remains a therapeutic challenge. The choice of therapy for HES should be carefully guided based on the balance between therapeutic benefit and potential for side effects. More research on safety of use of imatinib on a wide scale of patients with HES is needed.
